# Medical News

**Published:** 1856-05

**Authors:** 


### THE MEDICAL EXAMINER.

PHILADELPHIA, MAY, 1856.

### MEDICAL NEWS.

MEDICAL SOCIETY OF THE STATE OF PENNSYLVANIA, SESSION 1856.—The next session of the Medical Society of the State of Pennsylvania will be held in the city of Philadelphia commencing on Wednesday, May 28th, 1856, at 11 o'clock, A. M.

Delegates who arrive in the city on the preceding day are requested to report themselves on the afternoon of that day between 4 and 6 o'clock at the Hall of the College of Physicians, Spruce St. between 8th and 9th. The building to be occupied by the Society during its session will be designated in the papers of the day.

Reports and communications to be laid before the Society may be sent in advance to either of the Recording Secretaries.

HENRY CARPENTER, Lancaster.
ALFRED L. KENNEDY, Philada.

---

The following list comprises the graduates of our different Colleges during the last session, all, at least, that we have noticed in our exchanges. University of Pennsylvania, 140; Jefferson College, 215; Pennsylvania College, 37; Philadelphia College of Medicine, 19; University Medical College, N. Y., 97; New York Medical College, 32; College of Physicians and Surgeons, 39; University of Buffalo, "several;" Memphis Medical College, 11; Dartmouth College, 15;

Rush Medical College, 42; Medical College of Ohio, 17; Medical College of South Carolina, 85; Miami Medical College, 18; Cleveland Medical College, 38; Massachusetts Medical College, 28; Stearling Medical College, 20; University of Nashville, 85; University of Michigan, 30; University of Missouri, 28; St. Louis Medical College, 50; University of Louisiana, 65; Medical College of Georgia, 73; Medical College of Savannah, 12; Oglethorpe Medical College, 8. Total 1204, 411 of whom graduated in Philadelphia.

---

We ask the attention of our readers to Dr. Durkee's Case of Erythema Tuberculatum et Œdematosum, which will be found in the "Record" of this number. It contains a very admirable and particular account of a rare and malignant affection of the skin, but slightly mentioned by most dermatologists. We regret that our limited space will not permit us to extract the entire article.

---

We have received four numbers of the Cincinnati Medical Observer, edited by Geo. Mendenhall, M. D., J. A. Murphy, M. D., and Edward B. Stevens, M. D.; and two numbers of the Medical Independent, published at Detroit, and edited by H. Goadby, M. D., Ed. Kane, M. D., and L. G. Robinson, M. D. Both Journals give evidence in their pages of being well edited, and we willingly enter them upon our list of exchanges.

---

We are indebted to Dr. Betton, one of the Trustees of the State Lunatic Hospital at Harrisburg, for a copy of its fifth annual report. The report states that at the beginning of the year 1855 there were 214 patients in the Hospital, since which time 164 have been received and 128 discharged or died, leaving 250 under care. Of the discharged 26 were restored, 30 improved, 43 stationary, and 29 died. The highest number in the Hospital at any one time was 264.

For the proper working of the Institution during the present year, the board ask an appropriation from the State of $33,000. They also renew their suggestions for providing another State Institution west of the Alleghenies, as the present Hospital will soon prove incompetent to meet the demands upon it from the central and eastern parts of the State.

---

The French Academy of Science has conferred the prize of "Experimental Physiology" upon Dr. Brown Séquard, for his researches upon the Physiology of the Spinal Cord.

---

Dr. Chauncy Booth has been appointed Superintendent to the McLean Asylum, in place of Dr. Luther V. Bell, resigned.

